# Crispr/Cas9 Mediated Inactivation of Argonaute 2 Reveals its Differential Involvement in Antiviral Responses

**DOI:** 10.1038/s41598-017-01050-6

**Published:** 2017-04-21

**Authors:** Márta Ludman, József Burgyán, Károly Fátyol

**Affiliations:** Agricultural Biotechnology Institute, National Agricultural Research and Innovation Centre, Szent-Györgyi Albert u. 4, Gödöllő, 2100 Hungary

## Abstract

RNA silencing constitutes an important antiviral mechanism in plants. Small RNA guided Argonaute proteins fulfill essential role in this process by acting as executors of viral restriction. Plants encode multiple Argonaute proteins of which several exhibit antiviral activities. A recent addition to this group is AGO2. Its involvement in antiviral responses is established predominantly by studies employing mutants of *Arabidopsis thaliana*. In the virological model plant, *Nicotiana benthamiana*, the contribution of AGO2 to antiviral immunity is much less certain due to the lack of appropriate genetic mutants. Previous studies employed various RNAi based tools to down-regulate *AGO2* expression. However, these techniques have several disadvantages, especially in the context of antiviral RNA silencing. Here, we have utilized the CRISPR/Cas9 technology to inactivate the *AGO2* gene of *N. benthamiana*. The *ago2* plants exhibit differential sensitivities towards various viruses. AGO2 is a critical component of the plants’ immune responses against PVX, TuMV and TCV. In contrast, *AGO2* deficiency does not significantly influence the progression of tombusvirus and CMV infections. In summary, our work provides unequivocal proof for the virus-specific antiviral role of AGO2 in a plant species other than *A. thaliana* for the first time.

## Introduction

The discovery of nucleic acid guided nucleases (NGNs) has fundamentally influenced molecular biology in the last two decades. Directing nucleases to their substrates using the simple rules of Watson-Crick base pairing serves the basis for a number of gene regulatory and defensive mechanisms employed by both prokaryotes and eukaryotes. In eukaryotes, one of the best-studied NGN-based processes is RNA silencing (or RNA interference, RNAi), which is also a major antiviral defense mechanism in plants^1–7^. Antiviral RNA silencing is triggered by double-stranded RNA (dsRNA) replication intermediates of viruses or intramolecular fold-back structures within their genomes. These structures are processed by RNase III-like enzymes (DCLs), generating primary viral small interfering RNAs (vsiRNAs). Endogenous RNA-dependent RNA polymerases (RDRs) can also use single-stranded (ss) viral RNAs to synthesize dsRNAs, which serve as substrates for the production of secondary vsiRNAs. Both classes of vsiRNAs are eventually incorporated into Argonaute (AGO) protein containing RNA-induced silencing complexes (RISCs). RISCs can inhibit virus amplification either by directly slicing the viral genome or by limiting the expression of virus-encoded proteins in a cleavage-dependent or -independent fashion. While primary vsiRNAs limit virus replication predominantly locally, secondary vsiRNAs appear to play an important role in restricting systemic infection. Many viruses have evolved various means to evade detection and neutralization by the above mechanisms. One of their most effective countermeasures is the deployment of proteins - viral suppressors of RNA silencing (VSRs) - that interferes with RNA silencing at different steps^8^.

NGN-based mechanisms are also employed by prokaryotes to protect the integrity of their genome^9–11^. Adaptive immune systems, based on the concerted actions of clustered regularly interspaced short palindromic repeat (CRISPR) loci and CRISPR associated (Cas) endonucleases, help the elimination of invading genetic parasites such as viruses and plasmids in many species of archaea and eubacteria. At the end of 2012 a breakthrough paper reported a repurposed version of the type II CRISPR system from *Streptococcus pyogenes* ^9^. In the modified system, the Cas9 endonuclease is programmed with an engineered single guide RNA (sgRNA), allowing the cleavage of any 20 nt DNA sequence that lies 5′ to an NGG motif (PAM, protospacer adjacent motif). Soon after, it was shown that the SpCas9 protein could find and cleave its target sequence not only in bacteria but in the chromatin context of higher eukaryotes as well^12, 13^. The introduced double strand DNA break can be repaired either by homologous recombination (HR), if appropriate template is present, or more frequently by the error-prone non-homologous end-joining (NHEJ) pathway. Since then, due to its simplicity and robustness, the system has been successfully adapted for gene inactivation and genome editing in many organisms, including a number of plant and animal species^14–16^.

The genome of the model plant *Arabidopsis thaliana* encodes 10 AGO proteins, of which the antiviral roles of AGO1, AGO2, AGO4, AGO5, AGO7 and AGO10 have been demonstrated^17–19^. These analyses have been greatly facilitated by the availability of extensive collections of mutants. Although, *A. thaliana* is an excellent model organism for studying many aspects of plant physiology, it is a non-host for many viruses, considerably limiting its use for investigating plant-virus interactions. In stark contrast, *Nicotiana benthamiana* is exceptionally susceptible to a wide array of plant pathogens, including viruses, bacteria, oomycetes and fungi^20^. However up until now, its widespread use as a model organism has been greatly hampered by the fact that like many members of the *Nicotiana* genus, *N. benthamiana* has an amphidiploid genome. This feature significantly hiders the generation of genetic mutants of this plant. Importantly, the advent of the CRISPR/Cas9 technology can offer a solution for the above problem by allowing efficient production of mutants of this genetically intractable species.

In addition to *AGO1* and *AGO4*, recent data have also implicated *AGO2* in antiviral defenses of *N. benthamiana* ^21–25^. These findings however, were made in plants where *AGO2* expression was down-regulated by RNAi (VIGS, shRNA). The effectiveness of these techniques can sometimes be uncertain (especially in virus infected plants) and other issues may also complicate the interpretation of the obtained results (see later in discussion). To avoid these problems, we set out to generate *ago2* mutants of *N. benthamiana*, using the CRISPR/Cas9 system of *S. pyogenes*. The created mutant plants were challenged with a number of viruses. The susceptibility of the *ago2* plants to different viruses varied significantly. For the first time, conclusive proof was provided for the involvement of *AGO2* in antiviral immunity in a plant species other than *A. thaliana*.

## Materials and Methods

### Plasmid construction

Plasmid construction was performed using conventional techniques^26^. The bi-functional Cas9-sgRNA targeting plasmid was created as follows: (1) Plant codon optimized *SpCas9* gene was cloned into pENTR11. (2) The resulting plasmid was used to transfer the *SpCas9* gene into the pK7WG2D binary plasmid using LR clonase to yield pK7WG2D-SpCas9. (3) *A. thaliana* U6 promoter driven sgRNA expression cassette was assembled by over-lapping PCRs^27^. (4) After digesting with AatII-XhoI, the expression cassette was ligated into pK7WG2D-SpCas9, which was also cleaved with the same restriction enzymes. The *N. benthamiana* genome was searched for potential off target sites of the selected sgRNA using the CCTop-CRISPR/Cas9 target online predictor (http://crispr.cos.uni-heidelberg.de/^28^.

### Agroinfiltration, protein analysis

Agroinfiltration of *N. benthamiana* leaves and western blot analysis of protein lysates were carried out as described^24^.

### Plant transformation

Agrobacterium mediated leaf disc transformation of *N. benthamiana* was performed essentially as described^29^. Briefly, *N. benthamiana* plants were cultured under sterile conditions on MS medium for 2–3 months. Leaf discs were cut from the plants and infected with a C58C1 *A. tumefaciens* strain carrying the binary Cas9-sgRNA targeting construct. The infected discs were incubated on MS104 agar plates for two days. Next, the discs were transferred onto MS104 agar plates supplemented with 300 μg/ml kanamycin and 500 μg/ml cefotaxime. Every month the discs were passaged onto fresh selective plates. Approximately after three month, the appearing shoots were transferred onto rooting medium. Finally, plantlets with fully developed root system were transplanted into appropriate soil mix and reared under standard greenhouse conditions.

### Evaluation of CRISPR/Cas9 mediated target modification, identification of *ago2* mutant *N. benthamiana* plants

Efficiency of the selected *AGO2*-specific sgRNA was tested in transient agroinfiltration assays. Briefly, *N. benthamiana* leaves were infiltrated with a suspension of *Agrobacteria* carrying the binary Cas9-sgRNA targeting construct. Three days later, genomic DNA was extracted from the infiltrated leaf tissue using a DNeasy Plant Kit (Qiagen) according to the manufacturer’s instructions. The targeted region of the *AGO2* gene was PCR amplified using Phusion polymerase (Thermo). Subsequently, the 564 bp PCR product was cloned into pGEM-T easy plasmid vector (Promega). To assess target modification efficiency, 20 plasmid clones containing the *AGO2* fragment was sequenced.

To identify stable *N. benthamiana* transformant lines carrying CRISPR/Cas9 modified *AGO2* alleles, the targeted segment of the *AGO2* gene was PCR amplified using Phire Plant Direct PCR kit (Thermo). The PCR fragments were cloned into pGEM T easy plasmid. At least ten independent insert containing plasmid clones were obtained and sequenced from each transformant line to appraise their *AGO2* status.

### RNA analysis

Preparation of total RNA from leaf tissue and subsequent northern blot analyses were performed as described^24^.

### Virus inoculation

Cymbidium ringspot virus (CymRSV), carnation italian ringspot virus (CIRV) and turnip crinkle virus (TCV) inoculations were performed using *in vitro* transcribed full-length viral transcripts as described before^25^. Infections by GFP expressing turnip mosaic virus (TuMV-GFP) and potato virus X (PVX-GFP) were initiated either by infiltrating the appropriate *Agrobacterium* strains^30, 31^ into *N. benthamiana* leaves or by rubbing them with sap prepared from TuMV-GFP or PVX-GFP infected plants. PVX-GFP could also be transmitted via the root system by shared irrigation water. Cucumber mosaic virus (CMV) inoculations were carried out as described before^32^. Viral infection experiments were repeated at least three times. In every experiment the treatment groups consisted of three plants. The results were highly reproducible and representative data are shown. The protein and RNA samples, used for northern and western blots, were also prepared and pooled from the three plants, which constituted the given treatment group.

## Results

### Generation of *ago2* mutant *N. benthamiana*

**Figure 1:**
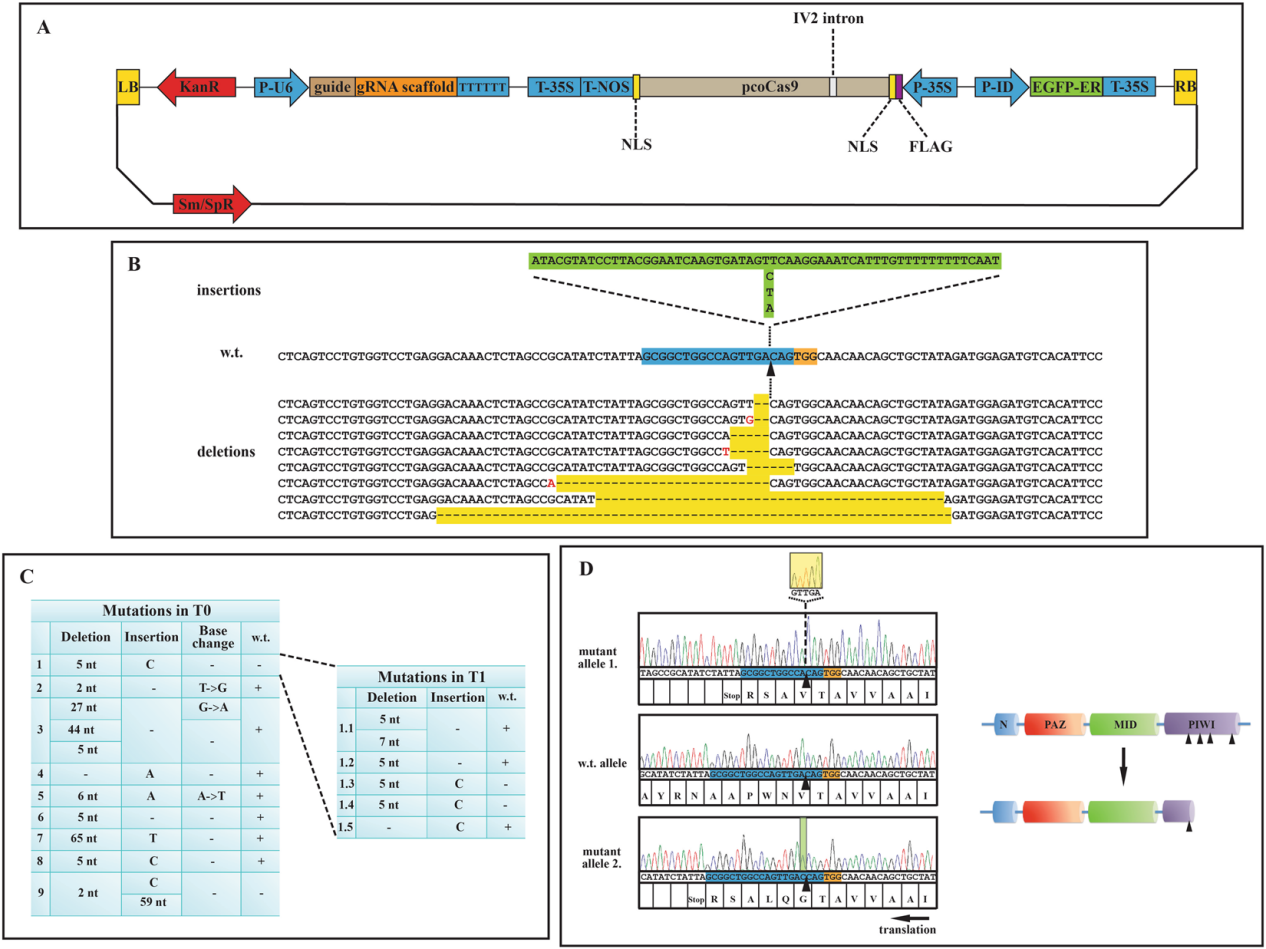
Generation of *ago2* mutant *N. benthamiana*. (**A**) Schematic structure of the bi-functional Cas9-sgRNA expression plasmid used for the targeted inactivation of the *AGO2* gene of *N. benthamiana*. (**B**) Structure of the isolated *ago2* alleles. The targeted sequence of the wild-type *AGO2* gene is highlighted with blue and the adjacent PAM motif with orange. In the isolated mutant alleles the insertions are highlighted with green and the deletions are with yellow. The nucleotide changes accompanying some of the deletions are indicated by red letters. (**C**) Summary of the mutations identified in the T0 and T1 transformant plants. (**D**) The mutations in the *ago2* alleles of transformant lines 1.3 and 1.4 result in premature stop codons. Both alleles encode truncated, dysfunctional AGO2 proteins, which lack three out of the four catalytically essential amino acids of the PIWI domain.

To examine the antiviral role of the AGO2 protein of *N. benthamiana*, we decided to generate *ago2* mutant plants using the type II CRISPR/Cas9 system of *S. pyogenes*. We created a single plasmid system to express the Cas9 protein and the appropriate sgRNA (Fig. 1A). With the use of such system, we hoped to achieve higher gene modification efficiency compared to multi-plasmid systems, where efficient co-delivery of the various components could be problematic. Despite its amphidiploid genome, transcriptome analyses have indicated that *N. benthamiana* expresses only a single *AGO2* homologue^33, 34^. An sgRNA, targeting the functionally essential PIWI domain of the expressed *AGO2* gene was designed. The CCTop plant CRISPR analysis online tool did not detect off target sites for this sgRNA in coding regions of *N. benthamiana*. The pK7WG2D binary plasmid vector was modified to co-express the sgRNA and the plant codon optimized SpCas9 nuclease^27^. Next, we tested the efficiency of the selected sgRNA in a transient assay system. *Agrobacteria* carrying the binary targeting construct was infiltrated into *N. benthamiana* leaves. Three days later, genomic DNA was extracted from the infiltrated leaf tissue and the targeted region of the *AGO2* gene was PCR amplified. The product was cloned into pGEM-T easy vector and the DNA fragments were sequenced. We detected Cas9 introduced mutations at the expected position in 6,25% of the analyzed DNA fragments (data not shown). The observed relatively low targeting efficiency was obviously an underestimate, since the leaf tissue used for genomic DNA purification comprised of a mixture of *Agrobacteria* infected and non-infected cells. Nonetheless, our results demonstrated that the CRISPR/Cas9 system was suitable to specifically target the *AGO2* gene of *N. benthamiana* using the designed sgRNA.

Next, we proceeded to generate stably transformed *N. benthamiana* plants using the well-established *Agrobacterium* mediated leaf disc transformation protocol^29^. Nine T0 transformant plants were regenerated on kanamycin containing medium and tested for the desired genetic modification. The targeted segment of the *AGO2* gene was PCR amplified, the products were cloned into pGEM-T easy plasmid and sequenced. Mutations in the *AGO2* gene were detected at the expected position in all transformants (Fig. 1B,C). Wild-type *AGO2* allele was also found in seven of the nine T0 plants. In five transformants more than two different *AGO2* alleles were found, indicating that these plants were chimeric. The identified mutations were mostly deletions starting at the predicted Cas9 cleavage site (3 nt upstream of the PAM sequence). The deletions ranged from 2 to 65 nt and extended in both 5′ and 3′ directions. Sometimes single nucleotide changes also accompanied the deletions. Less frequently insertions were also observed. These were mostly single nucleotide insertions at the Cas9 cleavage site. In one instance, insertion of a 59 nt DNA segment was also detected. BLAST search of this sequence revealed an identical region in the *N. benthamiana* genome. The DNA segment neither seemed to be a part of a functional coding region nor possessed any recognizable functional motif. Nonetheless, this finding indicated that in addition to the more conventional NHEJ and HR pathways, the double strand breaks generated by the Cas9 endonuclease could be sealed via the acquisition of pieces of DNA from distant parts of the genome.

Exclusively mutant *ago2* alleles were found in only two transformant lines (#1 and #9), however one of them (#9) carried three different *ago2* alleles. In transformant line #1 only two mutant *ago2* alleles were detected in our initial screen. Both the 5 nt deletion and the single C insertion (both at the Cas9 cleavage site) resulted in frame-shifts and as a consequence premature stop codons. Both alleles had the capacity to encode truncated AGO2 proteins, which lack three out of the four catalytically essential amino acid residues of the PIWI domain (E875, D907, D1037) (Fig. 1D). Earlier, we have demonstrated that the D907A mutation alone was sufficient to totally abolish substrate cleavage, gene silencing and antiviral activities of AGO2^24^. Consequently, the truncated AGO2 proteins encoded by the above *ago2* alleles were most likely dysfunctional. Transformant #1 was allowed to self-pollinate and T1 plants were generated. The targeted region of the *AGO2* gene was sequenced in five T1 plants, as described above. Three of the examined plants carried wild-type *AGO2* alleles, in addition to the mutant ones, indicating that the parental T0 plant was indeed chimeric. Importantly however, in lines #1.3 and #1.4 only two of the above-described *ago2* mutant alleles were detected. These biallelic plants were again allowed to self-pollinate and the resulting T2 progenies were used in all subsequent experiments as *ago2* mutant plants. The phenotype of these plants was indistinguishable from the parental wild-type *N. benthamiana* plants, under the growth conditions used in our experiments.

### *ago2* mutant *N. benthamiana* plants are hyper-susceptible to PVX infection

PVX is a type member of the *Potexvirus* genus (*Alphaflexiviridae*), which can predominantly infect Solanaceous hosts. Plants belonging to the *Brassicaceae* family are generally considered to be non-hosts for PVX. Paradoxically, most of the information on the mechanisms involved in restricting PVX infection has been derived from studies employing *A. thaliana*, which is naturally a non-host for this virus. Indeed, wild-type *A. thaliana* can not be infected by PVX. Efficient infection requires either the inactivation of *DCL*s (*DCL2* and/or *DCL4*) or *AGO2* ^35–37^. Recently, a synergistic role of *AGO5* in systemic PVX infection has also been reported^38^. Even though *N. benthamiana* is a natural host for PVX, almost nothing is known about the AGOs involved in the anti-PVX defenses of this plant. So far, we and others have only shown that in a transient over-expression system, the AGO2 protein (either from *N. benthamiana* or *A. thaliana*) has the capacity to locally limit the replication of a VSR and movement deficient form of PVX (PVX-ΔTGB)^24, 38^. However, the role of *AGO2* in a *bona fide* PVX infection in *N. benthamiana* has not been studied, due to the lack of the appropriate mutant. Thus, to investigate this question, we challenged our *ago2* and control wild-type *N. benthamiana* plants with a GFP expressing version of PVX^30^. The use of this virus allowed us to monitor the progression of infection *in vivo*. In most experiments, we initiated the infection from a leaf-infiltrated *Agrobacterium* strain carrying a PVX-GFP encoding binary vector. Inoculation of the plants by PVX virions either via the root system (irrigation) or the leaves (rubbing) provided essentially the same results.

**Figure 2:**
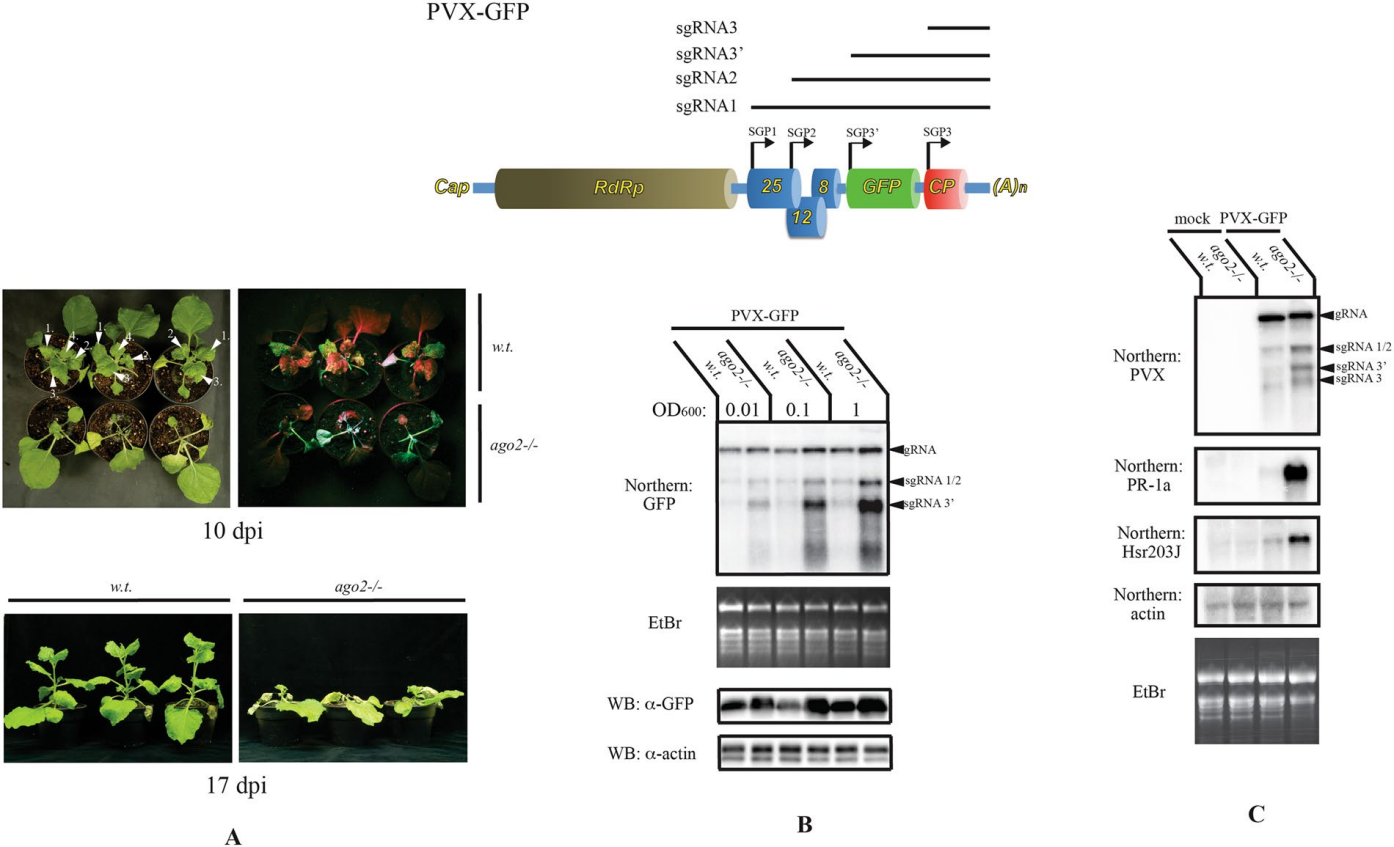
a*go2* mutant *N. benthamiana* plants are hyper-susceptible to PVX infection. (**A**) Photographs of PVX-GFP infected plants. As a sign of recovery new leaves were starting to emerge in wild-type plants, which were generally absent in *ago2* plants. On the top left panel white arrowheads point to newly emerging leaves, with higher numbers indicating younger leaves. Picture of the same plants was taken under UV light (top right panel). In the wild-type plants the new leaves exhibited gradually reduced GFP signals with decreasing age. At 17 dpi, wild-type plants show recovery, while in the *ago2* plants the apical necrosis usually results in plant death (bottom panel). (**B**) PVX infections of wild-type and *ago2 N. benthamiana* plants were initiated from a leaf-infiltrated *Agrobacterium* strain carrying a PVX-GFP encoding binary vector. Different dilutions of the bacterium suspension were infiltrated into the leaves. Concentrations of the suspensions are given as optical densities measured at 600 nm (OD_600_). At 5 dpi, total RNA and protein lysates were prepared from the first symptomatic non-inoculated leaves. Samples were collected from three plants and pooled. Viral RNA levels were monitored in northern blot using a GFP probe. Viral genomic and subgenomic RNAs are indicated by arrowheads (top panel). Ethidium-bromide (EtBr) stained gel is shown as loading control (second panel). Virus encoded GFP protein levels were analyzed by western blot (third panel). The same blot was developed with an actin antibody to verify equal loading (bottom panel). (**C**) Induction of defense related genes in PVX infected plants were monitored by northern blot. RNA samples were prepared from systemically infected leaves at 7 dpi. Samples were pooled from three plants. Actin probe hybridized filter and Ethidium-bromide stained gel are shown as loading controls. Arrowheads indicate PVX genomic and subgenomic RNAs.

Five days post-inoculation (dpi), signs of systemic infection could be observed on both wild-type and *ago2* plants. By 8–10 dpi, significant differences could be noted between the two cohorts (Fig. 2A). In wild-type plants, as a sign of recovery, new leaves were starting to emerge. With decreasing age, these leaves exhibited gradually reduced GFP signals, the youngest ones having only a few small fluorescent spots. In contrast, the mutant plants usually did not show any sign of recovery. Instead, necrosis of the apical stem region was visible, which generally culminated in the death of the plants by 17–21 dpi. Using a GFP specific probe, levels of virus RNA were also monitored in the inoculated plants by northern blot analysis (Fig. 2B). Despite the dramatically different outcome of infection, PVX genomic RNA (gRNA) levels were only slightly elevated in systemically infected leaves of the *ago2* mutants compared to wild-type plants. In contrast, subgenomic transcripts (sgRNAs) accumulated strongly in the mutant plants and the virally encoded GFP protein levels also largely followed the sgRNA levels (as assessed by western blot).

Virus induced systemic necrosis (SN) is accompanied by the up-regulation of defense-related genes^39^. In non-inoculated symptomatic leaves of PVX infected *ago2* mutants, northern blot analysis revealed robust induction of *PR-1a* and *Hsr203J* genes (both are reliable markers for virus induced SN), while their levels did not change significantly in wild-type plants (Fig. 2C). These results confirmed that PVX infection elicited a strong necrotic response in *ago2* plants.

### AGO2 is required for the recovery of *N. benthamiana* from TuMV infection

In *A. thaliana*, genetic analysis has identified the involvement of several AGO proteins in defense against TuMV (*Potyviridae*), in a modular and organ-specific manner^40^. Though highly susceptible, *N. benthamiana* can efficiently recover from TuMV infection. The genetic background of this recovery is largely unexplored. Nonetheless, two sets of observations have suggested that RNA silencing plays an essential role in the defense against potyviruses in this species as well:

1. Silencing of *RDR6* causes hyper-accumulation of potato virus Y (PVY) and plum pox virus (PPV) in *N. benthamiana* ^41, 42^. This indicates that RDR6-derived vsiRNAs are necessary for curtailing replication of potyviruses in this host.
2. Co-infection of *N. benthamiana* with PVX and PVY results in increased systemic symptoms (synergism) and eventually plant death. This synergism can be recapitulated by infecting the plants with a PVX chimera expressing the silencing suppressor of potyviruses (helper component proteinase, HC-Pro)^43^.

**Figure 3:**
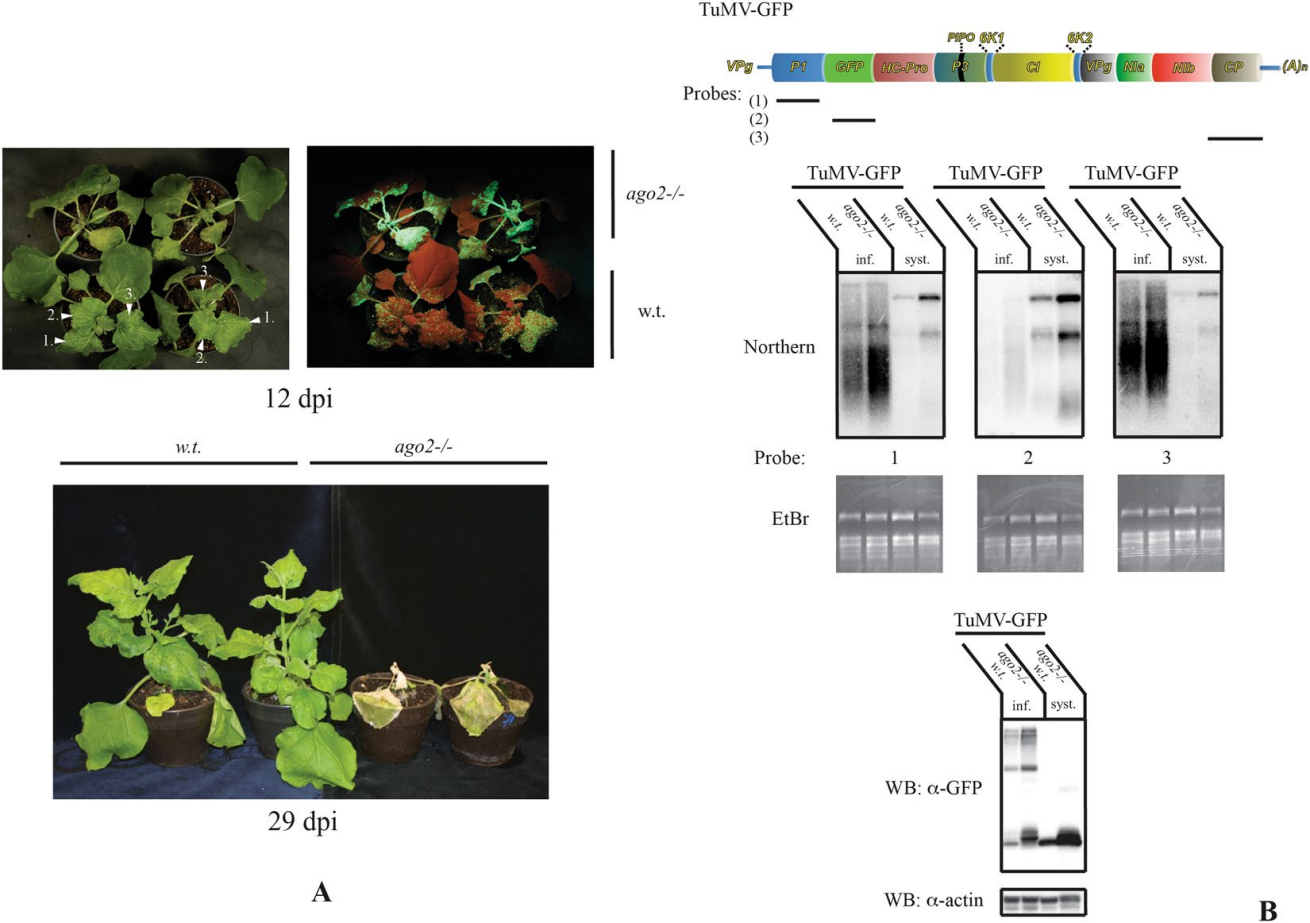
a*go2* mutant *N. benthamiana* plants are hyper-susceptible to TuMV infection. (**A**) Photographs of TuMV-GFP infected plants. On the top left panel white arrowheads indicate newly emerging leaves in the recovering wild-type plants. Higher numbers correspond to younger leaves. Top right panel shows picture of the same plants taken under UV light. Younger apical leaves exhibit decreased GFP accumulation. No signs of recovery were detectable in *ago2* plants. By 29 dpi, wild-type plants almost completely recovered, while *ago2* plants died (bottom panel). (**B**) TuMV infection was initiated in *N. benthamiana* plants from a leaf-infiltrated *Agrobacterium* strain carrying a TuMV-GFP encoding binary expression vector. At 8 dpi, total RNA and protein lysates were prepared from the infiltrated and first symptomatic systemically infected leaves. Samples were collected from three plants and pooled. Accumulation of TuMV viral RNA was analyzed in infiltrated and systemically infected leaves by northern blot using the indicated probes. Ethidium-bromide stained gels are shown as loading controls. Virus encoded GFP levels were monitored by western blot. The partially processed TuMV polyprotein species were detected by GFP antibody as high molecular weight signals in the infiltrated lysates. Equal loading was verified by actin antibody staining.

To evaluate the role of *AGO2* in TuMV infection, *ago2* mutant and wild-type *N. benthamiana* plants were inoculated with a GFP expressing form of TuMV (TuMV-GFP). The infection was usually initiated from a leaf infiltrated *Agrobacterium* strain, which carries a TuMV-GFP transgene encoding binary vector^31^. Inoculation of the plants with virion-containing sap was also performed and gave essentially the same results. As a sign of systemic infection, GFP signals were starting to emerge in apical non-inoculated leaves at 7–8 dpi in plants of both genetic backgrounds. By 12 dpi, the symptoms exhibited by the mutant and wild-type plants became clearly distinguishable (Fig. 3A). In wild-type plants, new leaves were starting to emerge, which exhibited fewer and fewer fluorescent foci. In contrast, in the mutants the apical regions became necrotic, with no signs of recovery. After four weeks, the wild-type plants almost completely recovered from the infection, while the *ago2* mutants died by that time. The amounts of TuMV-derived RNA and GFP protein were also monitored in the two groups of plants (Fig. 3B). In general, both accumulated to higher quantities in the inoculated and systemically infected leaves of *ago2* mutants compared to wild-type plants. In summary, the symptomatologies of PVX and TuMV infections were quite similar in *N. benthamiana* though, the latter proceeded with a somewhat delayed kinetics. The *AGO2* status of the plants critically influenced the outcome of infections by both viruses.

### *AGO2* deficiency exacerbates symptoms of TCV infection in *N. benthamiana*

The *Carmovirus* TCV has a broad host range, which predominantly includes members of the *Brassicaceae*. It can also infect *N. benthamiana* as an experimental host and is one of the relatively few viruses that are highly virulent on *A. thaliana*. An earlier study has demonstrated that *ago1* and *ago7* mutants are hypersusceptible to TCV infection^44^. Recently, it has also been shown that *AGO2* plays a critical role in the survival of TCV infected *A. thaliana* ^45, 46^. The role of various AGOs in TCV infection in *N. benthamiana* is unexplored. RNA silencing is most likely involved in the process, since infection of this host with a chimeric PVX construct, expressing the VSR of TCV (the coat protein of TCV, TCV-CP), elicits severe systemic tissue necrosis and subsequent death by 7–10 dpi^47^. Interestingly, the symptoms of TCV infection are relatively mild on *N. benthamiana*, which include vein clearing, leaf curling and shortened internodes. Generally, no lethality is associated with the infection. The apparent contradiction between the above observations can be reconciled by the finding that the essential determinant of the silencing suppressor activity of TCV (the N terminal 25 amino acids of TCV-CP) is inaccessible within the native virion capsid structure.

**Figure 4:**
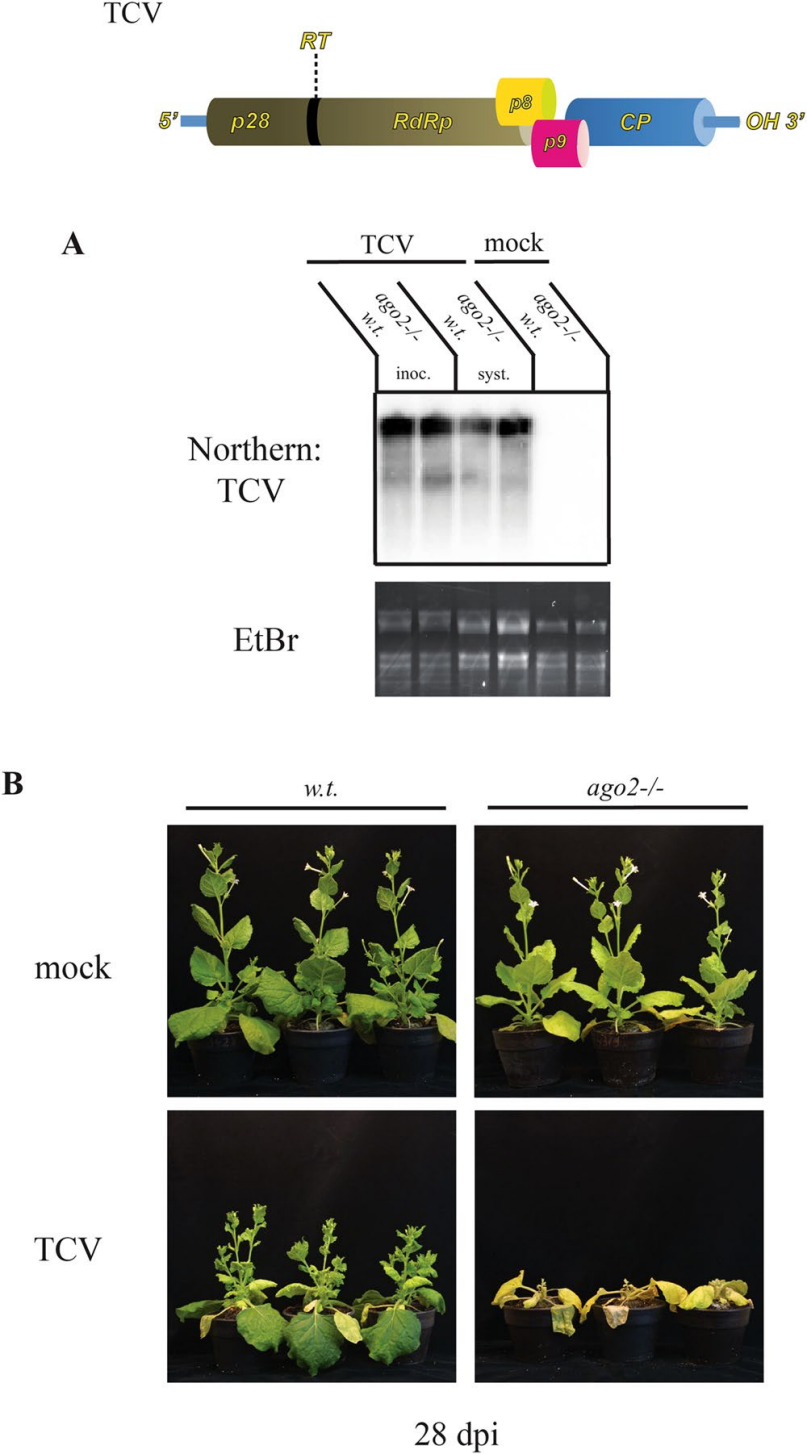
AGO2 deficiency exacerbates symptoms of TCV infection in *N. benthamiana*. (**A**) Wild-type and *ago2 N. benthamiana* plants were infected with *in vitro* transcribed full-length TCV viral transcripts. As controls, mock infections were also performed. Accumulation of TCV genomic RNA was analyzed in inoculated and systemically infected leaves at 7 dpi by northern blot. As a loading control the Ethidium-bromide stained gel is shown. (**B**) Photographs of TCV and mock infected wild-type and *ago2* plants were taken at 28 dpi.

To assess the effect of *AGO2* deficiency on TCV infection in *N. benthamiana*, *ago2* and wild-type control plants were inoculated with *in vitro* transcribed infectious TCV RNA. gRNA levels were monitored in the inoculated and non-inoculated symptomatic leaves of the infected plants by northern blotting (Fig. 4A). At 7 dpi, similar buildups of gRNAs were detected in both cohorts. Symptoms of infection were starting to diverge between the two groups at 14 dpi. In the mutant plants, extension of the apical internodes was strongly reduced compared to the wild-type ones, however unlike in PVX or TuMV infected *ago2* plants, no apical necrosis was detectable. By 28 dpi, the mutant plants became severely chlorotic and usually died, while the wild-type controls only exhibited the moderate symptoms of TCV infection (vein clearing, leaf curling and shortened internodes) (Fig. 4B).

### *AGO2* has a modulatory effect on tombusvirus infection

**Figure 5:**
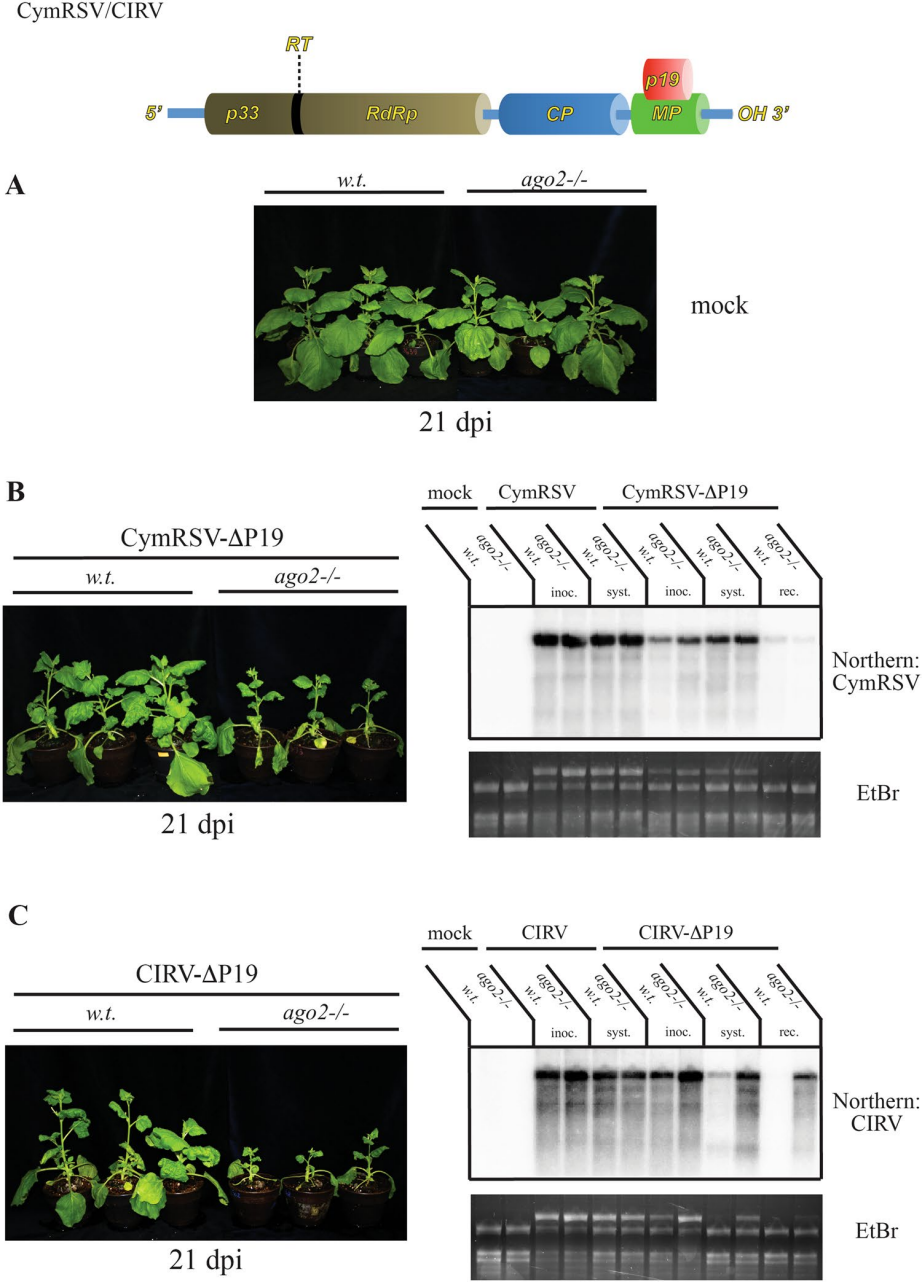
Effect of AGO2 on tombusvirus infection. Wild-type and *ago2 N. benthamiana* plants were infected with *in vitro* transcribed wild-type and ΔP19 mutant CymRSV (**B**) and CIRV (**C**) viral transcripts. Mock infections were also performed (**A**). At 5 dpi, total RNA was prepared from the inoculated and the first symptomatic non-inoculated leaves. RNA samples were purified from recovered apical leaves at 21 dpi. Samples were collected from three plants and pooled. Viral gRNA accumulation was monitored by northern blot. Ethidium-bromide stained gels are shown as loading controls. Photographs of virus infected and mock infected *N. benthamiana* plants are also shown at 21 dpi.

Tombusviruses are well suited to study antiviral RNA silencing because they produce large quantities of vsiRNAs during infection. In addition, they encode a 19 kD protein (P19), which is one of the most potent and best studied suppressors of RNA silencing^25, 48–50^. Although, a wealth of biochemical data is available on the antiviral mechanisms elicited by tombusviruses, genetic dissection of the process is greatly hampered by the fact that *A. thaliana* is a non-host for these pathogens. *N. benthamiana AGO2* has been implicated in the defense against tomato bushy stunt virus (TBSV). However, these data have been obtained on plants, in which *AGO2* expression was only partially knocked-down by VIGS or shRNA^22, 23^. Thus, we tested the sensitivity of our *ago2* mutants against various tombusviruses. Wild-type CymRSV and CIRV caused very severe systemic symptoms in both wild-type and mutant plants, which generally resulted in their death by 7–10 dpi (data not shown). The genetic backgrounds of the plants had no effect on the progression of the disease. This was also reflected in the comparable accumulation of viral gRNAs in the inoculated and systemically infected leaves of the two groups of plants (Fig. 5).

Tombusviral P19 is a strong suppressor of RNA silencing and may be able to mask the antiviral activity of *AGO2*. To avoid the interference by P19, the above infections were repeated with VSR deficient forms of CymRSV and CIRV (CymRSV-ΔP19 and CIRV-ΔP19). These viruses elicited much milder symptoms in both wild-type and *ago2* plants. No lethality was detected in either groups and signs of recovery became obvious by 2 weeks after inoculation. By 21 dpi, wild-type plants mostly recovered. Although somewhat stunted, clear signs of recovery were also exhibited by the *ago2* mutants. Interestingly however, some reproducible differences could be noted between the infection kinetics of CymRSV-ΔP19 and CIRV-ΔP19. The recovery of the *ago2* plants infected with the former virus proceeded faster than the ones infected with the latter. The patterns of gRNA accumulation were also different between the two viruses (Fig. 5). CymRSV-ΔP19 gRNA reached similar levels in the inoculated and systemically infected leaves of both wild-type and mutant plants. At 21 dpi, gRNAs were barely detectable in either groups. In contrast, CIRV-ΔP19 gRNA accumulated to higher levels in *ago2* mutants than in wild-type plants, which was especially pronounced in systemically infected leaves. The difference persisted at least until 3 weeks after infection.

Recently, a critical role for *AGO2* has been reported during the progression of TBSV-ΔP19 infection in *N. benthamiana* ^22, 23^. Thus, we inoculated *ago2* and wild-type plants with *in vitro* transcribed infectious TBSV-ΔP19 viral transcript. Symptom development and viral RNA levels were followed as above. The pattern of TBSV-ΔP19 RNA accumulation was similar to that of CIRV-ΔP19. Likewise, the recovery of *ago2* plants was only slightly delayed compared to wild-type controls (Suppl. Fig. 1). The difference between our results and the ones reported earlier, was likely due to the different methods employed for the down-regulation of *AGO2* (RNAi vs. CRISPR mediated mutagenesis).

### Progression of CMV infection is not significantly affected by *AGO2* deficiency in *N. benthamiana*

**Figure 6:**
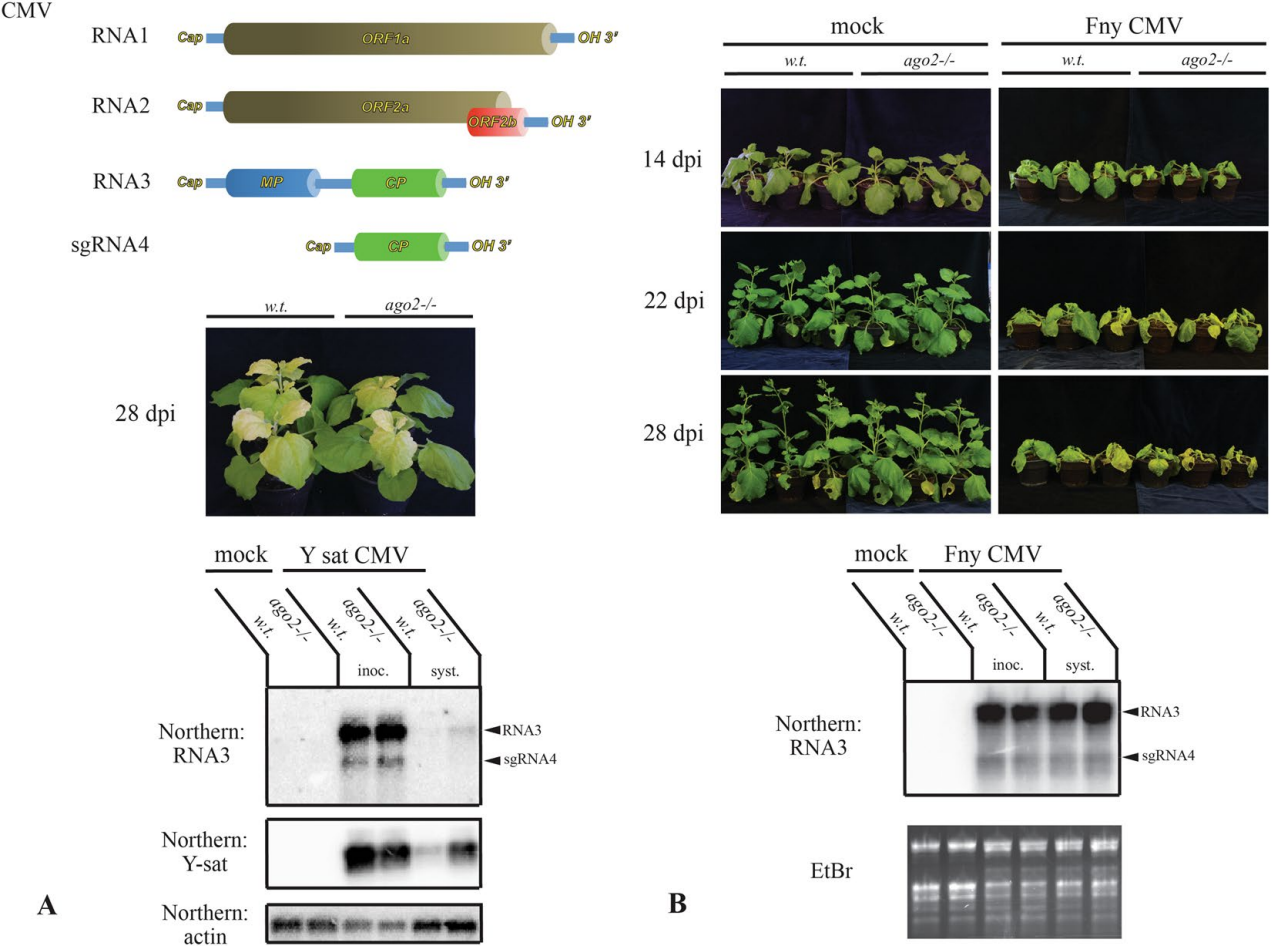
CMV infection is not affected by AGO2 deficiency in *N. benthamiana*. (**A**) Wild-type and *ago2 N. benthamiana* plants were infected with sap produced from Y-sat CMV infected *N. benthamiana*. Photographs of infected plants were taken at 28 dpi (top panel). At 14 dpi, RNA was prepared from the inoculated and the first systemically infected leaves. Samples were prepared from three plants and pooled. Accumulations of CMV RNA3 and sgRNA4 were monitored by northern blot using an RNA3-specific probe (second panel). Arrowheads indicate CMV RNA3 and sgRNA4. Y-sat levels were also analyzed by northern blot (third panel). The same filter was also hybridized with an actin probe as a loading control (bottom panel). (**B**) Wild-type and *ago2 N. benthamiana* plants were infected with sap produced from Fny CMV infected *N. benthamiana*. Mock infections were also performed with sap of uninfected plants. To monitor the progression of CMV infection, photographs of infected plants were taken at 14 dpi, 22 dpi and 28 dpi. At 7 dpi, RNA was prepared from the inoculated and the first symptomatic non-inoculated leaves. Samples were taken from three plants and pooled. Accumulations of CMV RNA3 and sgRNA4 were monitored by northern blot using an RNA3-specific probe. Arrowheads indicate CMV RNA3 and sgRNA4. Ethidium-bromide stained gel is shown as loading control.

CMV (*Bromoviridae*) is a tripartite plus stranded RNA virus with a wide host range. Study of the infection process by CMV provided one of the first examples of the antiviral role of RNA silencing in plants^51, 52^. The involvements of *AGO1*, *AGO2* and *AGO4* in anti-CMV defenses have been extensively demonstrated in *A. thaliana* ^19, 45, 53–56^. To evaluate the role of *AGO2* in CMV infection in *N. benthamiana, ago2* and wild-type plants were inoculated with the Y-sat strain of CMV. Y-sat (Y satellite RNA) is a noncoding subviral RNA, which can modify the symptoms of CMV infection in certain hosts^32, 57^. It generates siRNAs, which down-regulate the mRNA of a key enzyme of chlorophyll biosynthesis (magnesium protoporphyrin chelatase subunit I, *ChlI*) in *N. tabacum* and *N. benthamiana*, thereby resulting in a characteristic yellow phenotype. The use of this virus-satellite combination allowed us to easily monitor the progression of virus infection. Signs of systemic infection (yellowing of the apical leaves) started to appear at the same time (12–14 dpi) in both groups of plants and even at 28 dpi, no significant difference could be noted between the two genotypes (Fig. 6A). RNA3 of CMV exhibited comparable accumulation in the inoculated leaves of both wild-type and *ago2* plants. In the systemically infected leaves, only slightly higher viral RNA level could be detected in *ago2* mutants relative to the wild-type plants. Y-sat was present in similar quantities in the inoculated leaves of both groups. However, in the systemically infected leaves of *ago2* mutants, Y-sat accumulated to considerably higher level than in the equivalent leaves of wild-type plants. The significance of this observation remains to be investigated.

Virus associated satellite and defective interfering (DI) RNAs can significantly modify symptom manifestation during virus infection^58^. Therefore, the surprising lack of effect of *AGO2* on the progression of CMV infection might be due to the presence of the Y satellite. To inspect this question more closely, the highly aggressive Fny strain of CMV (which is not associated with any satellite RNA) was used to infect wild-type and *ago2* plants, instead of the temperate Y-sat strain. Again, the two cohorts exhibited nearly identical symptoms until 28 dpi (Fig. 6B). No difference in the accumulation of RNA3 could be noted either between the two plant genotypes. In summary, the progression of CMV infection did not seem to be significantly influenced by *AGO2* in *N. benthamiana*.

## Discussion

Programmable DNA cleavage by CRISPR/Cas9 enables targeted inactivation of genes and it also serves the basis for more sophisticated genome engineering. This system has been demonstrated to work efficiently not only in single cells, but in whole organisms as well. In genetically intractable species, like the amphidiploid *N. benthamiana*, the use of this technology opens up avenues to generate mutants, which can be used for the genetic dissection of various biological phenomena. Thus far, in species like *N. benthamiana* largely RNAi based methodologies (antisense RNA, VIGS, shRNA) have been available to interrupt the functions of genes of interest. Although widely accepted, skeptics often point out that these techniques suppress gene expression indirectly and the knockdown is often only partial. Additionally, studying components of RNA silencing by using RNAi techniques presents the paradoxical situation, where one relies on the activity of those molecules, which he wishes to disable. Viral vectors applied for VIGS experiments (TRV, PVX etc.) may also interfere with various endogenous cellular functions and cause unintended changes in the expression of non-target genes, making it difficult to unambiguously interpret the results^59^. This can become especially problematic for studying host-virus interactions, since viruses often exhibit synergistic or antagonistic relationships with each other. Further complicating the issue is that viruses often encode proteins, which can inhibit RNA silencing (VSRs). To circumvent this problem, VSR deficient forms of viruses are frequently employed. However, the immense amounts of vsiRNAs generally produced during viral infections can themselves saturate and thereby neutralize the silencing effectors.

To circumvent the above problems, we used the CRISPR/Cas9 system to generate *ago2* mutants of *N. benthamiana*. Using a specific sgRNA, mutations were introduced into the catalytically essential PIWI domain of AGO2. In a single transformation, we were able to generate plants with both alleles of the expressed *AGO2* gene disrupted. During plant regeneration, chimera formation was frequently observed. However, repeated self-pollination rapidly produced plants with exclusively mutant *ago2* alleles (T2 generation). The mutated alleles contained premature stop codons and as a consequence encoded truncated, dysfunctional AGO2 proteins. Stable inheritance of the mutations in subsequent generations was demonstrated (followed up to T3, Suppl. Fig. 2). Under normal growth conditions, the *ago2* plants did not exhibit any obvious phenotypic alteration. However, *AGO2* deficiency had a significant impact on the plant’s antiviral immunity, a feature reproducibly observed in successive generations of independent transformant lines (#1 and #9). Segregation of the *Cas9* transgene from the induced mutation did not alter the phenotype either. Additionally, a transformant line carrying the *Cas9* transgene on its own behaved indistinguishably from the non-transformed, parental *N. benthamiana* plants (Suppl. Fig. 3). Combined, these data confirmed that the observed phenotype of the *ago2* plants was the consequence of the inactivation of the *AGO2* gene and independent of the integration site of the T-DNA or the presence of the *Cas9* transgene.

The lack of AGO2 had differential effects on the plants’ antiviral responses, upon which the viruses could be categorized into two groups:

1. AGO2 was a critical component of the antiviral response. The viruses that belonged to this group (PVX, TuMV and TCV) were quite dissimilar and the symptoms they elicited were also diverse. The lack of AGO2 resulted in apical necrosis and eventual lethality in both PVX and TuMV infected plants. Contrary, during TCV infection no localized necrosis was evident, however after 4 weeks general exacerbation of symptoms resulted in the death of the *ago2* plants.
2. AGO2 had no or only a minor, modulatory effect on virus infection. This group included various tombusviruses and CMV. The *AGO2* status of the plants did not influence the infection process caused by wild-type tombusviruses and only slightly affected the rate of recovery from infections elicited by their VSR deficient forms. These results are in agreement with a recent report suggesting that AGO1 is the main antiviral AGO involved in tombusvirus infection^25^. The symptoms of CMV infection on wild-type and *ago2* mutant *N. benthamiana* plants were essentially indistinguishable.

How one can explain the differential need for *AGO2* in the plant’s defensive measures elicited by various viruses? We hypothesize that the importance of *AGO2* in a specific antiviral response may reflect its reliance on the use of secondary vsiRNAs. The critical contribution of RDR6-derived secondary vsiRNAs in PVX, potyvirus and TCV provoked antiviral responses has already been demonstrated^41, 42, 44^. Contrary, their role in tombusvirus infection seems to be negligible^60^. The lack of effect of *AGO2* on CMV infection is however unexpected, as numerous studies have reported on the sensitizing effect of *AGO2* mutation on CMV infection and symptom manifestation in *A. thaliana* ^19, 45, 55^. A possible explanation can be related to the observation that in *N. benthamiana* the down-regulation of *RDR6* has not aggravated CMV infection^41^. This either suggests that secondary vsiRNAs do not play a critical role in anti-CMV immunity or more likely, other *RDR*s and/or *AGO*s (*RDR2*, *AGO1*, *AGO5* etc.) may have overtaken the above functions of *AGO2* and *RDR6* in this plant species. Regardless, the surprising lack of effect of *AGO2* on CMV infection in *N. benthamiana*, cautions against the simple extension of findings made in *A. thaliana* and emphasizes the need to employ additional model organisms to study host-virus interactions.

In summary, by using the CRISPR/Cas9 system we were able to generate an *ago2* mutant line of *N. benthamiana*, and for the first time provided unequivocal proof for the essential role of *AGO2* in antiviral immunity in a plant species other than *A. thaliana*. Most viruses are able to efficiently overcome the defense responses of their host plants. Thus, antiviral activities of components of RNA silencing can usually be studied using mutant viruses, which lack their cognate VSRs. However, VSRs are often multifunctional proteins involved not only in suppressing RNA silencing, but in many other aspects of the viral lifecycle. Therefore, their inactivation may result in viruses that are unable to carry out normal infection. The defenses that are effective against such crippled viruses may not be able to restrict the replication of wild-type ones. In this respect, it is especially significant that we were able to demonstrate the antiviral activity of *AGO2* against a collection of highly diverse wild-type viruses including PVX, TuMV and TCV.

## Electronic supplementary material

Supplemetary info-KF

## References

[1] Wilson RC, Doudna JA (2013). Molecular mechanisms of RNA interference. Ann Rev Biophys.

[2] Voinnet O (2009). Origin, biogenesis, and activity of plant microRNAs. Cell.

[3] Martinez de Alba AE, Elvira-Matelot E, Vaucheret H (2013). Gene silencing in plants: a diversity of pathways. Biochim Biophys Acta.

[4] Ding SW (2010). RNA-based antiviral immunity. Nat Rev Immunol.

[5] Carthew RW, Sontheimer EJ (2009). Origins and Mechanisms of miRNAs and siRNAs. Cell.

[6] Baulcombe D (2004). RNA silencing in plants. Nature.

[7] Csorba T, Kontra L, Burgyan J (2015). viral silencing suppressors: Tools forged to fine-tune host-pathogen coexistence. Virology.

[8] Burgyan J, Havelda Z (2011). Viral suppressors of RNA silencing. Trends Plant Sci.

[9] Jinek M (2012). A programmable dual-RNA-guided DNA endonuclease in adaptive bacterial immunity. Science.

[10] Doudna JA, Charpentier E (2014). Genome editing. The new frontier of genome engineering with CRISPR-Cas9. Science.

[11] Hochstrasser ML, Doudna JA (2015). Cutting it close: CRISPR-associated endoribonuclease structure and function. Trends Biochem Sci.

[12] Cong L (2013). Multiplex genome engineering using CRISPR/Cas systems. Science.

[13] Mali P (2013). RNA-guided human genome engineering via Cas9. Science.

[14] Harrison MM, Jenkins BV, O’Connor-Giles KM, Wildonger J (2014). A CRISPR view of development. Genes Dev.

[15] Barrangou R, Doudna JA (2016). Applications of CRISPR technologies in research and beyond. Nat Biotech.

[16] Wang H, La Russa M, Qi LS (2016). CRISPR/Cas9 in Genome Editing and Beyond. Ann Rev Biochem.

[17] Mallory A, Vaucheret H (2010). Form, function, and regulation of ARGONAUTE proteins. Plant Cell.

[18] Poulsen C, Vaucheret H, Brodersen P (2013). Lessons on RNA silencing mechanisms in plants from eukaryotic argonaute structures. Plant Cell.

[19] Carbonell A, Carrington JC (2015). Antiviral roles of plant ARGONAUTES. Curr Opin Plant Biol.

[20] Goodin MM, Zaitlin D, Naidu RA, Lommel SA (2008). Nicotiana benthamiana: its history and future as a model for plant-pathogen interactions. Mol Plant Microbe Interact.

[21] Bhattacharjee S (2009). Virus resistance induced by NB-LRR proteins involves Argonaute4-dependent translational control. Plant J.

[22] Scholthof HB (2011). Identification of an ARGONAUTE for antiviral RNA silencing in Nicotiana benthamiana. Plant Physiol.

[23] Odokonyero D (2015). Transgenic down-regulation of ARGONAUTE2 expression in Nicotiana benthamiana interferes with several layers of antiviral defenses. Virology.

[24] Fatyol K, Ludman M, Burgyan J (2016). Functional dissection of a plant Argonaute. Nucleic Acids Res.

[25] Kontra L (2016). Distinct Effects of p19 RNA Silencing Suppressor on Small RNA Mediated Pathways in Plants. PLoS Pathog.

[26] Maniatis, T., Fritsch, E. F. & Sambrook, J. *Molecular Cloning: A Laboratory Manual*. (Cold Spring Harbor Laboratory Press, 1982).

[27] Li JF (2013). Multiplex and homologous recombination-mediated genome editing in Arabidopsis and Nicotiana benthamiana using guide RNA and Cas9. Nat Biotech.

[28] Stemmer M, Thumberger T, Del Sol Keyer M, Wittbrodt J, Mateo JL (2015). CCTop: An Intuitive, Flexible and Reliable CRISPR/Cas9 Target Prediction Tool. PloS One.

[29] Clemente, T. in *Agrobacterium Protocols* Vol. 1 *Methods in Molecular Biology* (ed K. Wang) 143–154 (Humana Press, 2006).

[30] Peart JR, Cook G, Feys BJ, Parker JE, Baulcombe DC (2002). An EDS1 orthologue is required for N-mediated resistance against tobacco mosaic virus. Plant J.

[31] Garcia-Ruiz H (2010). Arabidopsis RNA-dependent RNA polymerases and dicer-like proteins in antiviral defense and small interfering RNA biogenesis during Turnip Mosaic Virus infection. Plant Cell.

[32] Shimura H (2011). A viral satellite RNA induces yellow symptoms on tobacco by targeting a gene involved in chlorophyll biosynthesis using the RNA silencing machinery. PLoS Pathog.

[33] Nakasugi K (2013). De novo transcriptome sequence assembly and analysis of RNA silencing genes of Nicotiana benthamiana. PloS One.

[34] Nakasugi K, Crowhurst R, Bally J, Waterhouse P (2014). Combining transcriptome assemblies from multiple de novo assemblers in the allo-tetraploid plant Nicotiana benthamiana. PloS One.

[35] Andika IB (2015). Differential contributions of plant Dicer-like proteins to antiviral defences against potato virus X in leaves and roots. Plant J.

[36] Andika IB (2015). Different Dicer-like protein components required for intracellular and systemic antiviral silencing in Arabidopsis thaliana. Plant Signal Behav.

[37] Jaubert M, Bhattacharjee S, Mello AF, Perry KL, Moffett P (2011). ARGONAUTE2 mediates RNA-silencing antiviral defenses against Potato virus X in Arabidopsis. Plant Physiol.

[38] Brosseau C, Moffett P (2015). Functional and Genetic Analysis Identify a Role for Arabidopsis ARGONAUTE5 in Antiviral RNA Silencing. Plant Cell.

[39] Komatsu K (2010). Viral-induced systemic necrosis in plants involves both programmed cell death and the inhibition of viral multiplication, which are regulated by independent pathways. Mol Plant Microbe Interact.

[40] Garcia-Ruiz H (2015). Roles and programming of Arabidopsis ARGONAUTE proteins during Turnip mosaic virus infection. PLoS Pathog.

[41] Schwach F, Vaistij FE, Jones L, Baulcombe DC (2005). An RNA-dependent RNA polymerase prevents meristem invasion by potato virus X and is required for the activity but not the production of a systemic silencing signal. Plant Physiol.

[42] Vaistij FE, Jones L (2009). Compromised virus-induced gene silencing in RDR6-deficient plants. Plant Physiol.

[43] Pruss G, Ge X, Shi XM, Carrington JC, Bowman Vance V (1997). Plant viral synergism: the potyviral genome encodes a broad-range pathogenicity enhancer that transactivates replication of heterologous viruses. Plant Cell.

[44] Qu F, Ye X, Morris TJ (2008). Arabidopsis DRB4, AGO1, AGO7, and RDR6 participate in a DCL4-initiated antiviral RNA silencing pathway negatively regulated by DCL1. Proc Natl Acad Sci USA.

[45] Harvey JJ (2011). An antiviral defense role of AGO2 in plants. PloS One.

[46] Zhang X, Zhang X, Singh J, Li D, Qu F (2012). Temperature-dependent survival of Turnip crinkle virus-infected arabidopsis plants relies on an RNA silencing-based defense that requires dcl2, AGO2, and HEN1. J Virol.

[47] Thomas CL, Leh V, Lederer C, Maule AJ (2003). Turnip crinkle virus coat protein mediates suppression of RNA silencing in Nicotiana benthamiana. Virology.

[48] Vargason JM, Szittya G, Burgyan J, Hall TM (2003). Size selective recognition of siRNA by an RNA silencing suppressor. Cell.

[49] Lakatos L, Szittya G, Silhavy D, Burgyan J (2004). Molecular mechanism of RNA silencing suppression mediated by p19 protein of tombusviruses. EMBO J.

[50] Scholthof HB (2006). The Tombusvirus-encoded P19: from irrelevance to elegance. Nat Rev Microbiol.

[51] Mourrain P (2000). Arabidopsis SGS2 and SGS3 genes are required for posttranscriptional gene silencing and natural virus resistance. Cell.

[52] Vaucheret H, Beclin C, Fagard M (2001). Post-transcriptional gene silencing in plants. J Cell Sci.

[53] Zhang X (2006). Cucumber mosaic virus-encoded 2b suppressor inhibits Arabidopsis Argonaute1 cleavage activity to counter plant defense. Genes Dev.

[54] Gonzalez I (2010). Cucumber mosaic virus 2b protein subcellular targets and interactions: their significance to RNA silencing suppressor activity. Mol Plant Microbe Interact.

[55] Wang XB (2011). The 21-nucleotide, but not 22-nucleotide, viral secondary small interfering RNAs direct potent antiviral defense by two cooperative argonautes in Arabidopsis thaliana. Plant Cell.

[56] Hamera S, Song X, Su L, Chen X, Fang R (2012). Cucumber mosaic virus suppressor 2b binds to AGO4-related small RNAs and impairs AGO4 activities. Plant J.

[57] Smith NA, Eamens AL, Wang MB (2011). Viral small interfering RNAs target host genes to mediate disease symptoms in plants. PLoS Pathog.

[58] Palukaitis P (2016). Satellite RNAs and Satellite Viruses. Mol Plant Microbe Interact.

[59] Olah E, Pesti R, Taller D, Havelda Z, Varallyay E (2016). Non-targeted effects of virus-induced gene silencing vectors on host endogenous gene expression. Arch Virol.

[60] Szittya G (2010). Structural and functional analysis of viral siRNAs. PLoS Pathog.

