# Supplementary material for: Crispr/Cas9 Mediated Inactivation of Argonaute 2 Reveals its Differential Involvement in Antiviral Responses
Source: Sci Rep. 2017 Apr 21;7:1010. doi: 10.1038/s41598-017-01050-6 (PMC5430636; doi:10.1038/s41598-017-01050-6)
Supplement: Supplementary file 1 — Supplemetary info-KF [file 41598_2017_1050_MOESM1_ESM.pdf]

CRISPR/CAS9 MEDIATED INACTIVATION OF ARGONAUTE 2 REVEALS ITS DIFFERENTIAL INVOLVEMENT IN ANTIVIRAL RESPONSES

Márta Ludman, József Burgyán*, Károly Fátyol*

Agricultural Biotechnology Institute, National Agricultural Research and Innovation Centre, Szent-Györgyi Albert u. 4. Gödöllő 2100 Hungary

*Correspondence:

Károly Fátyol

H-2101 Gödöllő, P. O. Box. 411, Hungary

TEL: 36-28-526-100

E-mail: kfatyol@gmail.com

József Burgyán

H-2101 Gödöllő, P. O. Box. 411, Hungary

TEL: 36-28-526-100

E-mail: burgyan@abc.hu

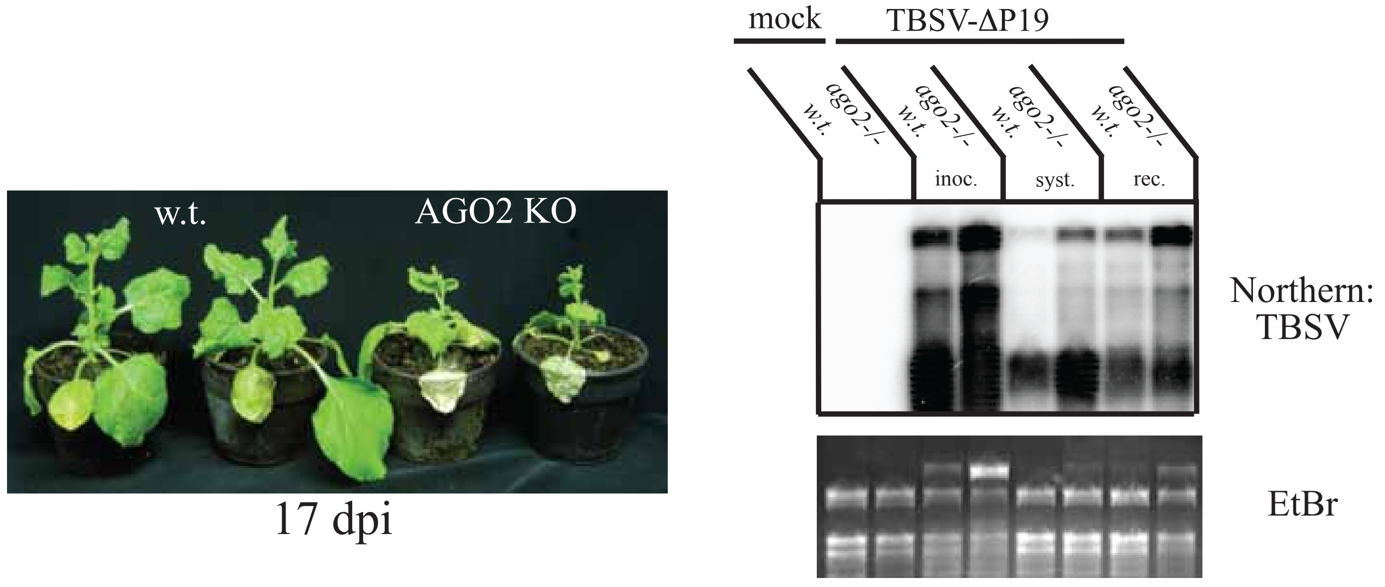

Suppl. Figure 1.

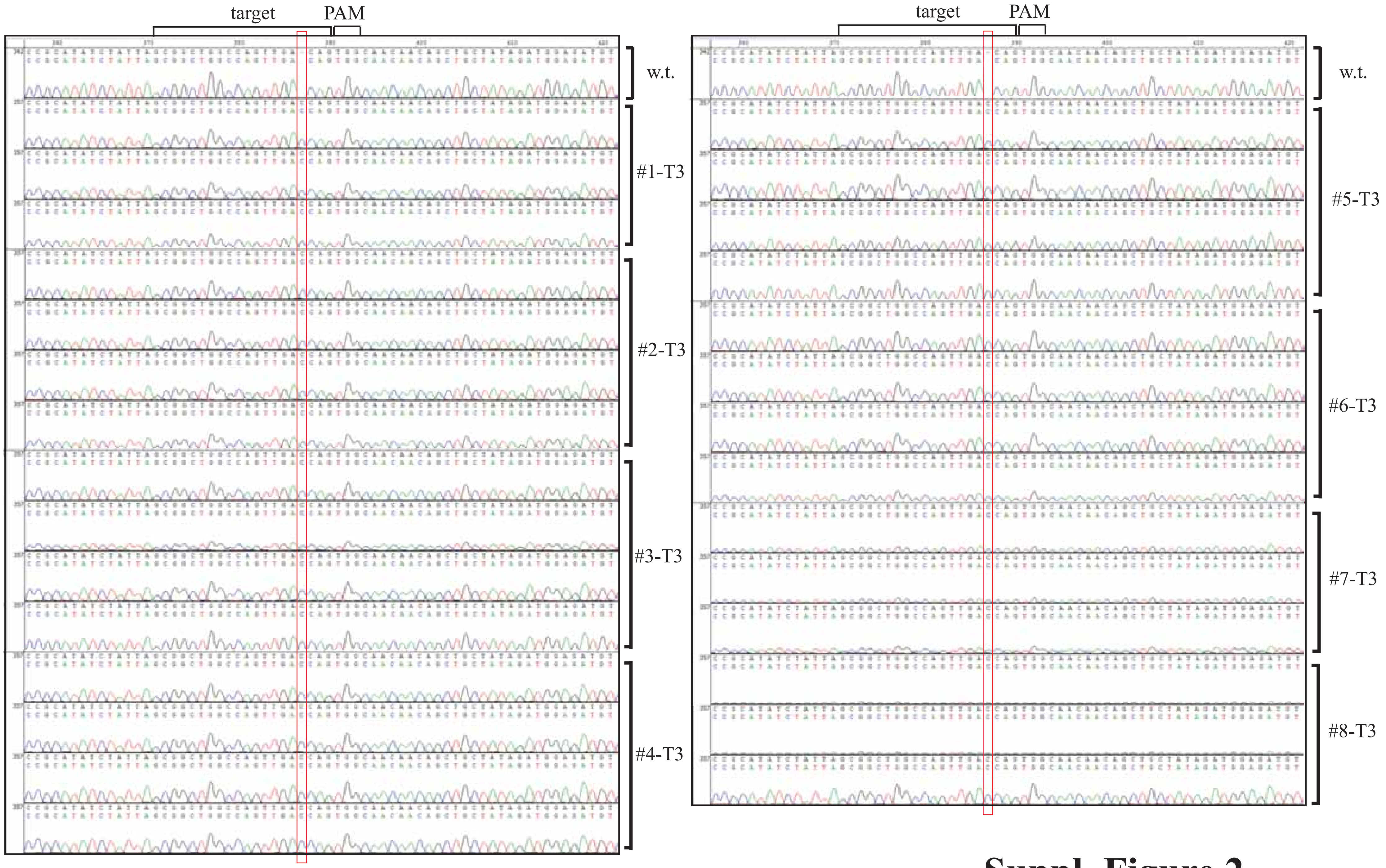

Suppl. Figure 2.

Cas9 trangenic plants

T3 *ago2* plants

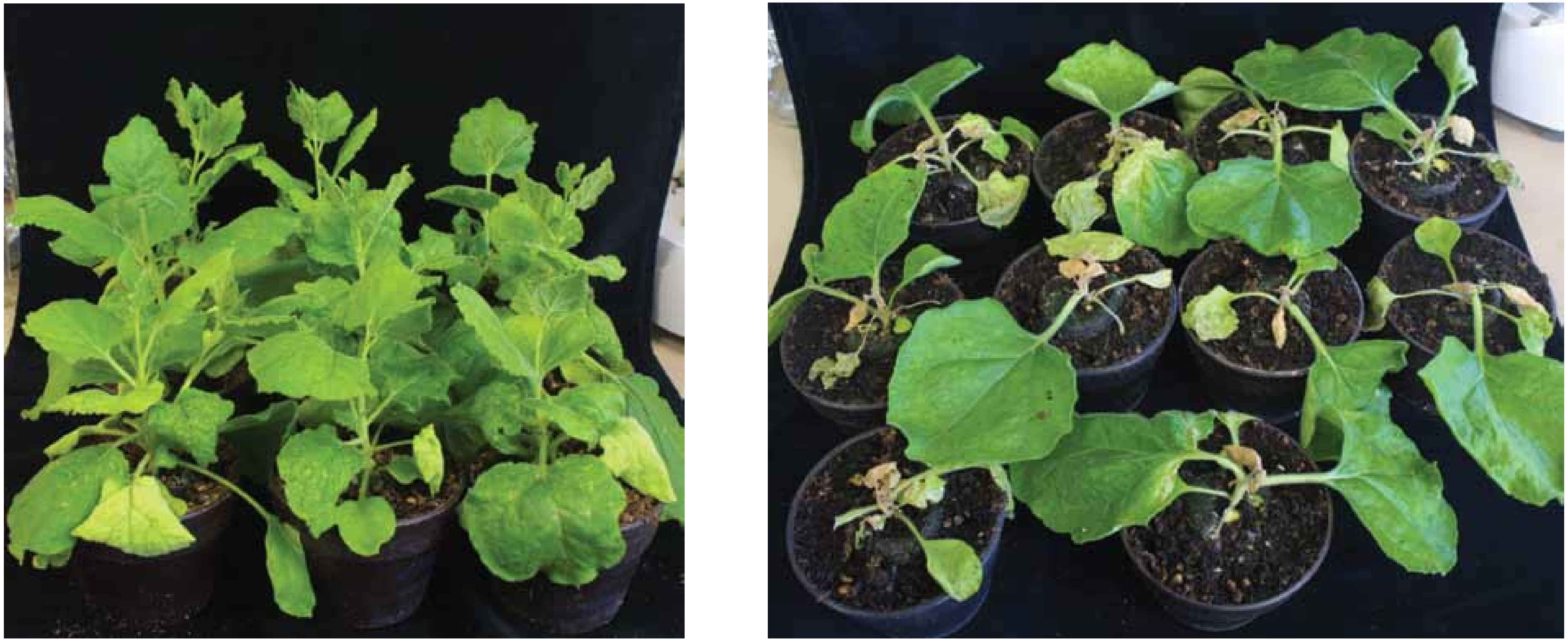

16 dpi

Suppl. Figure 3.

SUPPLEMENTARY FIGURE LEGENDS

Supplementary Figure 1. Effect of *AGO2* on TBSV-ΔP19 infection.
Wild-type and *ago2 N. benthamiana* plants were infected with *in vitro* transcribed TBSV-ΔP19 viral transcripts. At 5 dpi, total RNA was prepared from the inoculated and the first symptomatic systemically infected leaves. RNA samples were also purified from recovered apical leaves at 17 dpi. Samples were collected from three plants and pooled. Viral gRNA accumulation was monitored by northern blot. Ethidium-bromide stained gels are shown as loading controls. Photographs of virus infected *N. benthamiana* plants are also shown at 17 dpi.

Supplementary Figure 2. Stable inheritance of CRISPR/Cas9 induced *AGO2* mutations.
The targeted segment of the *AGO2* gene was PCR amplified, the products were cloned into pGEM-T easy plasmid and sequenced. Sequence chromatograms of the targeted *AGO2* region of eight T3 *ago2* plants are shown. Three or four plasmids per plant were sequenced. The single C insertion was detected in each sample and boxed in red. Chromatogram of the corresponding wild-type *AGO2* sequence is shown on top. The sgRNA target sequence and the adjacent PAM motif are also indicated.

Supplementary Figure 3. Increased virus sensitivity is a stable feature of *ago2 N. benthamiana* and is independent of the *Cas9* transgene.
Transgenic *N. benthamiana* plants carrying the Cas9 transgene on its own (left) and T3 *ago2* mutant *N. benthamiana* plants (right) were infected with PVX-GFP. Photographs of virus-infected plants were taken at 16 dpi. T3 *ago2* plants exhibit apical necrosis, while Cas9 transgenic plants can efficiently recover from PVX infection similarly to non-transgenic wild-type *N. benthamiana*.
